# Supplementary material for: Essential Role of BMP4 Signaling in the Avian Ceca in Colorectal Enteric Nervous System Development
Source: Int J Mol Sci. 2023 Oct 27;24(21):15664. doi: 10.3390/ijms242115664 (PMC10650322; doi:10.3390/ijms242115664)
Supplement: Supplementary file 1 [file ijms-24-15664-s001.zip › ijms-2615205-supplementary.pdf]

Supplemental figures

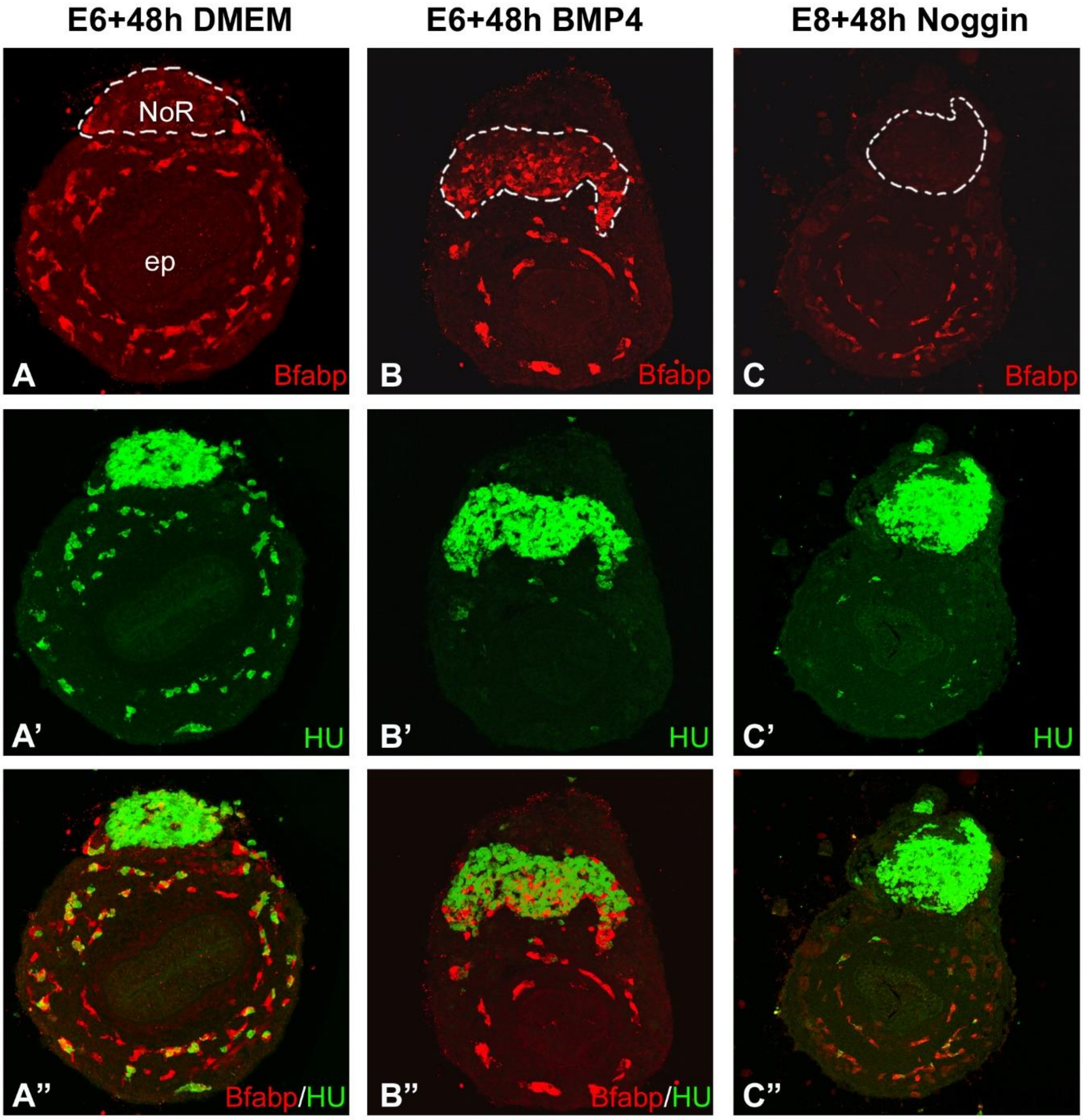

Figure S1. BMP4 overexpression enhances gliogenesis. E6 chick hindgut with ceca was cultured for 2 days with or without BMP4 or Noggin protein. Consecutive sections from cultured hindguts were stained for HU and Bfabp, which are markers of glial and neuronal differentiation, respectively (A-C'), show that HU+ neurons are present in all treatment groups, while Bfabp is increased following BMP4 treatment (B-B") and decreased following Noggin treatment (C-C"). *ep, epithelium; NoR, nerve of Remak.*

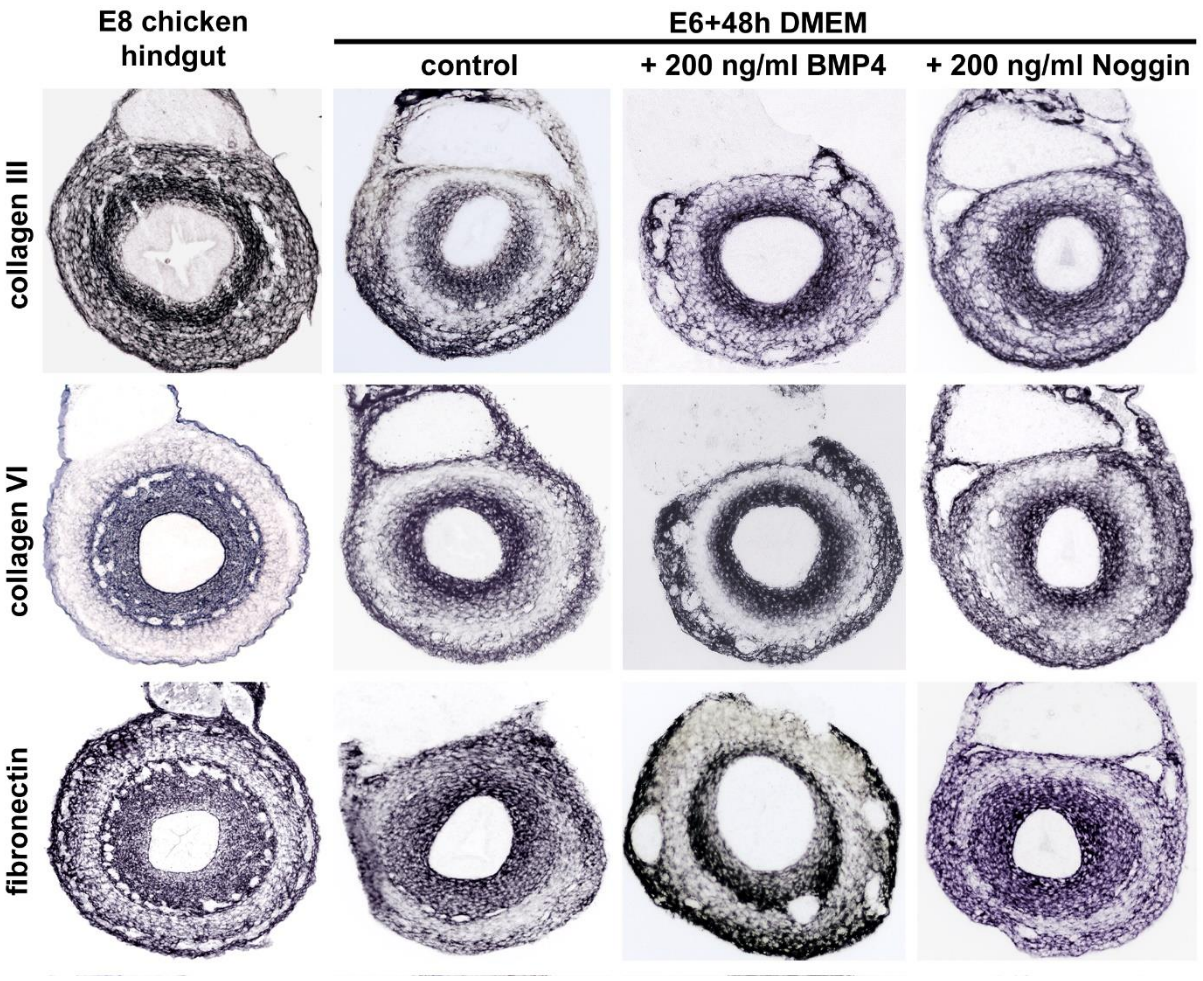

Figure S2. Expression of ECM proteins by immunohistochemistry. Consecutive 12 μm sections of wild-type E8 chick embryonic hindgut, E6+48 h cultured hindgut, E6+48 h BMP4 and Noggin treated hindgut. The expression of collagen III, collagen VI and fibronectin in transverse sections through the mid-hindgut is shown.

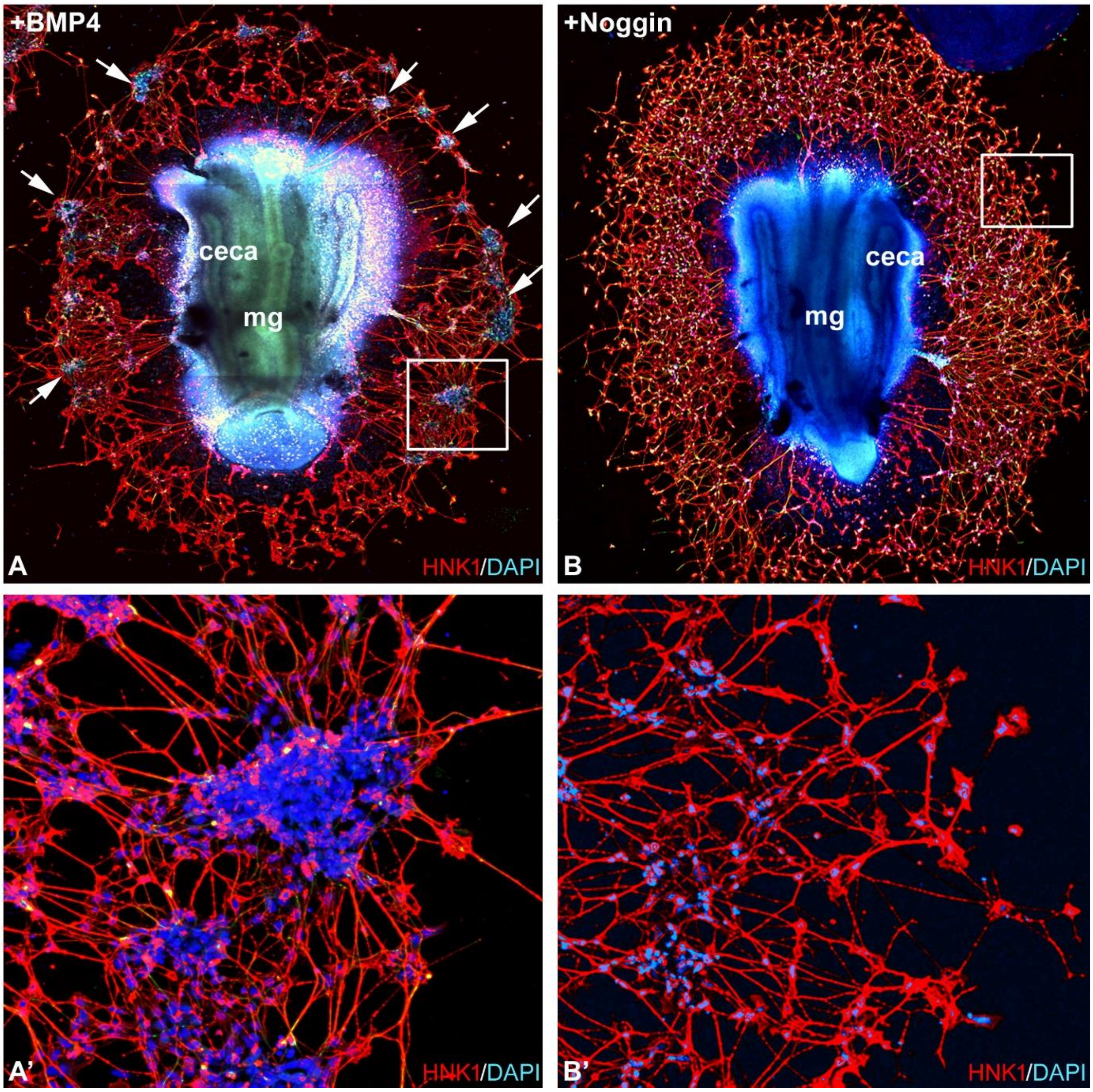

Figure S3. The presence of BMP4 induces a significant increase in cell aggregation. E6 ceca were cultured on fibronectin-coated plates with GDNF for 24 h followed by 24 h with either BMP4 (A) or addition of Noggin protein (B) and stained with HNK1 antibody to assess ENCDC migration and differentiation. Magnified view in inset (A',B') demonstrates that the addition of BMP4 induce robust HNK1+ cell aggregation (A, arrows). *mg, midgut*
